# Supplementary material for: Exploring the Potential of High-Molar-Activity Samarium-153 for Targeted Radionuclide Therapy with [153Sm]Sm-DOTA-TATE
Source: Pharmaceutics. 2022 Nov 23;14(12):2566. doi: 10.3390/pharmaceutics14122566 (PMC9785812; doi:10.3390/pharmaceutics14122566)
Supplement: Supplementary file 1 [file pharmaceutics-14-02566-s001.zip › pharmaceutics-2012258-supplementary.pdf]

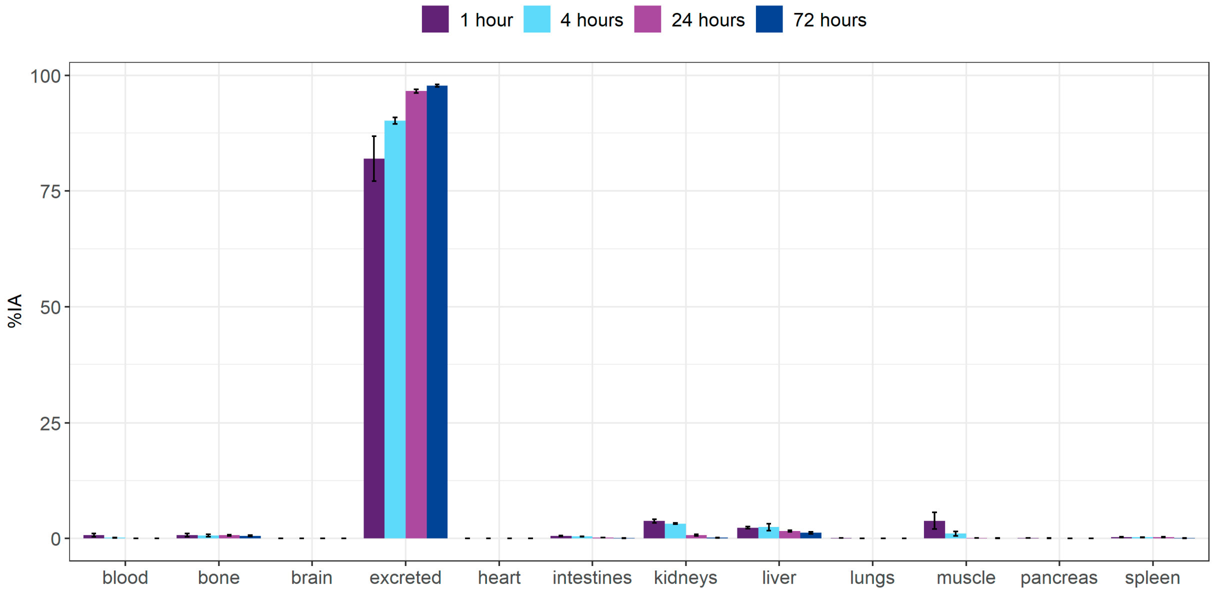

Supplementary Figure S1. Ex vivo biodistribution over time expressed as percentage of injected activity (%IA) of [^{153}Sm]Sm-DOTA-TATE in BALB/c mice. Values represent mean ± standard deviation (*n* = 3-4).

Supplementary Table S1. Biodistribution data expressed as percentage of injected activity (%IA)* of [^{153}Sm]Sm-DOTA-TATE in male BALB/c mice. Values represent mean ± standard deviation (*n* = 3-4).

	%IA			
	**1 h**	**4 h**	**24 h**	**72 h**
Blood	0.7 ± 0.3	0.2 ± 0.0	0.0 ± 0.0	0.0 ± 0.0
Bone	0.8 ± 0.4	0.6 ± 0.2	0.7 ± 0.1	0.6 ± 0.1
Brain	0.0 ± 0.0	0.0 ± 0.0	0.0 ± 0.0	0.0 ± 0.0
Excreted	82.0 ± 4.8	90.2 ± 0.7	96.6 ± 0.4	97.8 ± 0.2
Heart	0.0 ± 0.0	0.0 ± 0.0	0.0 ± 0.0	0.0 ± 0.0
Intestines	0.6 ± 0.1	0.5 ± 0.0	0.2 ± 0.0	0.1 ± 0.0
Kidneys	3.8 ± 0.4	3.2 ± 0.1	0.7 ± 0.2	0.2 ± 0.0
Liver	2.34 ± 0.2	2.5 ± 0.8	1.6 ± 0.2	1.2 ± 0.2
Lungs	0.1 ± 0.0	0.0 ± 0.0	0.0 ± 0.0	0.0 ± 0.0
Muscle	3.8 ± 1.8	1.1 ± 0.5	0.1 ± 0.0	0.1 ± 0.0
Pancreas	0.1 ± 0.0	0.1 ± 0.0	0.0 ± 0.0	0.0 ± 0.0
Spleen	0.3 ± 0.1	0.3 ± 0.0	0.3 ± 0.1	0.1 ± 0.0
Stomach	0.2 ± 0.0	0.1 ± 0.1	0.0 ± 0.0	0.0 ± 0.0

* Percentage of injected activity calculated as (cpm in organ/total cpm) × 100

Supplementary Table S2. Biodistribution data expressed as standard uptake value (SUV)* of [^{153}Sm]Sm-DOTA-TATE in male BALB/c mice. Values represent mean ± standard deviation (*n* = 3-4).

	SUV			
	**1 h**	**4 h**	**24 h**	**72 h**
Blood	0.1 ± 0.0	0.0 ± 0.0	0.0 ± 0.0	0.0 ± 0.0
Bone	0.1 ± 0.0	0.1 ± 0.0	0.1 ± 0.0	0.1 ± 0.0
Brain	0.0 ± 0.0	0.0 ± 0.0	0.0 ± 0.0	0.0 ± 0.0
Heart	0.1 ± 0.0	0.0 ± 0.0	0.0 ± 0.0	0.0 ± 0.0
Kidneys	2.1 ± 0.2	1.8 ± 0.1	0.4 ± 0.1	0.1 ± 0.0
Liver	0.4 ± 0.1	0.5 ± 0.2	0.3 ± 0.0	0.2 ± 0.0
Lungs	0.2 ± 0.0	0.1 ± 0.0	0.0 ± 0.0	0.0 ± 0.0
Muscle	0.6 ± 0.3	0.2 ± 0.1	0.0 ± 0.0	0.0 ± 0.0
Pancreas	0.2 ± 0.0	0.1 ± 0.0	0.0 ± 0.0	0.0 ± 0.0
Spleen	0.8 ± 0.2	0.8 ± 0.2	0.9 ± 0.3	0.3 ± 0.1

SUV = Standardized uptake value. Calculated as (radioactivity in cpm in organ/weight of organ in grams) / total cpm recovered/body weight).

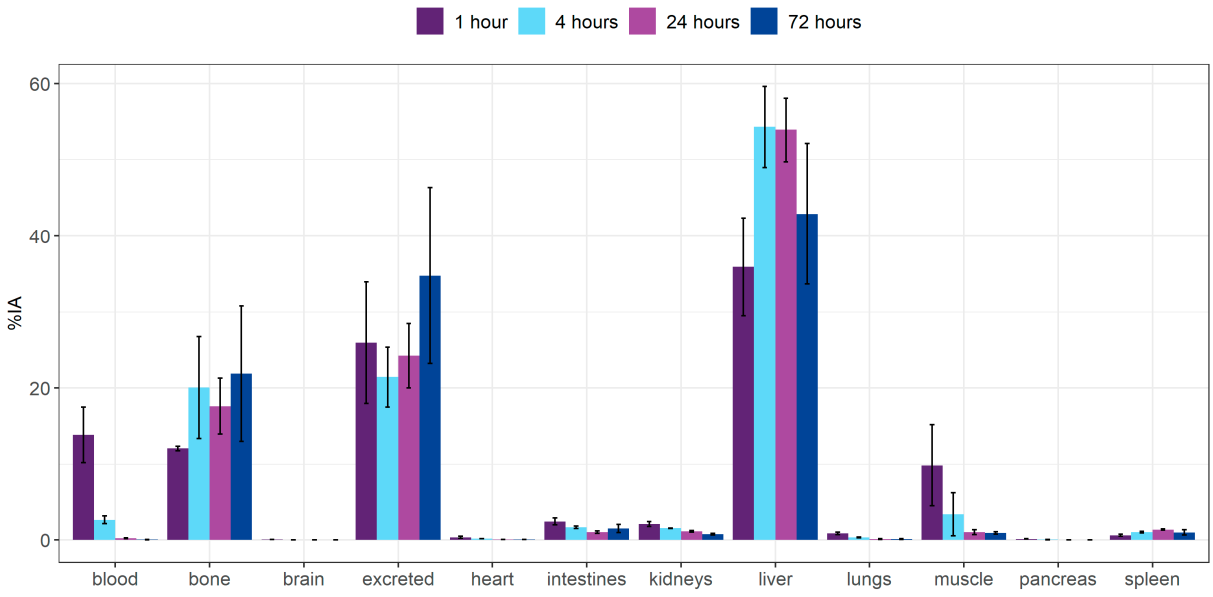

Supplementary Figure S2. Ex vivo biodistribution over time expressed as percentage of injected activity (%IA) of [^{153}Sm]$SmCl_3$ in BALB/c mice. Values represent mean ± standard deviation (*n* = 3-4).

Supplementary Table S3: Biodistribution data time expressed as percentage of injected activity (%IA)* of [^{153}Sm]SmCl$_3$ in male BALB/c mice. Values represent mean ± standard deviation (n = 3-4).

	%IA			
	**1 h**	**4 h**	**24 h**	**72 h**
Blood	13.8 ± 3.6	2.7 ± 0.5	0.2 ± 0.0	0.0 ± 0.0
Bone	12.1 ± 0.3	20.1 ± 6.7	17.6 ± 3.7	21.9 ± 8.9
Brain	0.1 ± 0.0	0.0 ± 0.0	0.0 ± 0.0	0.0 ± 0.0
Excreted	26.0 ± 8.0	21.4 ± 3.9	24.2 ± 4.2	34.8 ± 11.6
Heart	0.4 ± 0.1	0.2 ± 0.0	0.1 ± 0.0	0.1 ± 0.0
Intestines	2.4 ± 0.5	1.7 ± 0.2	1.0 ± 0.2	1.5 ± 0.5
Kidneys	2.1 ± 0.3	1.6 ± 0.0	1.1 ± 0.1	0.8 ± 0.1
Liver	35.9 ± 6.4	54.3 ± 5.3	53.9 ± 4.2	42.9 ± 9.2
Lungs	0.9 ± 0.2	0.3 ± 0.0	0.1 ± 0.0	0.1 ± 0.0
Muscle	9.8 ± 5.3	3.4 ± 2.8	1.0 ± 0.3	0.9 ± 0.2
Pancreas	0.1 ± 0.0	0.0 ± 0.0	0.0 ± 0.0	0.0 ± 0.0
Spleen	0.6 ± 0.1	1.0 ± 0.1	1.4 ± 0.1	1.0 ± 0.3
Stomach	0.5 ± 0.2	0.2 ± 0.0	0.2 ± 0.2	0.2 ± 0.1

* Percentage of injected activity calculated as (cpm in organ/total cpm) x 100.

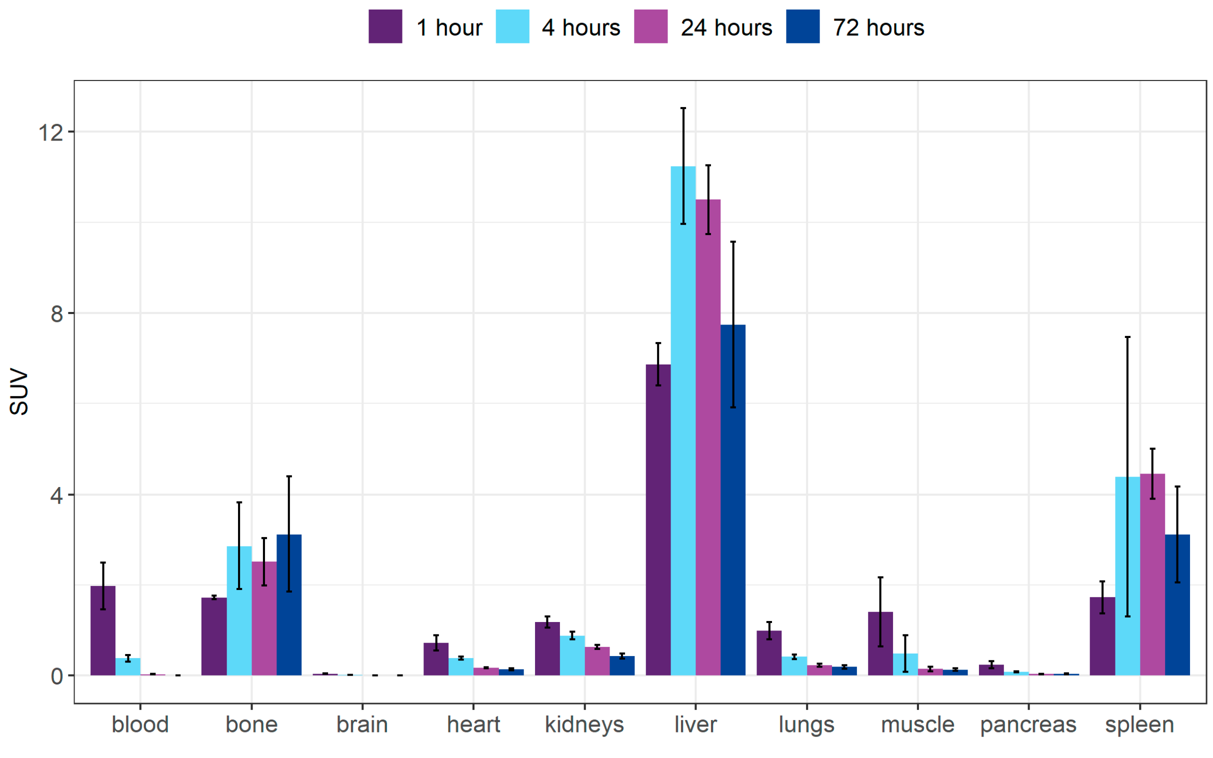

Supplementary Figure S3. Ex vivo biodistribution over time expressed as standard uptake value (SUV) of [^{153}Sm]SmCl$_3$ in BALB/c mice. Values represent mean ± standard deviation (n = 3-4).

Supplementary Table S4: Biodistribution data expressed as standard uptake value (SUV)* of [^{153}Sm]SmCl$_3$ in male BALB/c mice. Values represent mean ± standard deviation (n = 3-4).

	SUV			
	**1 h**	**4 h**	**24 h**	**72 h**
Blood	2.0 ± 0.5	0.4 ± 0.1	0.0 ± 0.0	0.0 ± 0.0
Bone	1.7 ± 0.0	2.9 ± 1.0	2.5 ± 0.5	3.1 ± 1.3
Brain	0.0 ± 0.0	0.0 ± 0.0	0.0 ± 0.0	0.0 ± 0.0
Heart	0.7 ± 0.2	0.4 ± 0.0	0.2 ± 0.0	0.1 ± 0.0
Kidneys	1.2 ± 0.1	0.9 ± 0.1	0.6 ± 0.0	0.4 ± 0.0
Liver	6.9 ± 0.5	11.2 ± 1.3	10.5 ± 0.8	7.7 ± 1.8
Lungs	1.0 ± 0.2	0.4 ± 0.0	0.2 ± 0.0	0.2 ± 0.0
Muscle	1.4 ± 0.8	0.5 ± 0.4	0.1 ± 0.0	0.1 ± 0.0
Pancreas	0.2 ± 0.1	0.1 ± 0.0	0.0 ± 0.0	0.0 ± 0.0
Spleen	1.7 ± 0.4	4.4 ± 3.1	4.4 ± 0.6	3.1 ± 1.1

* SUV = Standardized uptake value. Calculated as (radioactivity in cpm in organ/weight of organ in grams) / total cpm recovered/body weight).

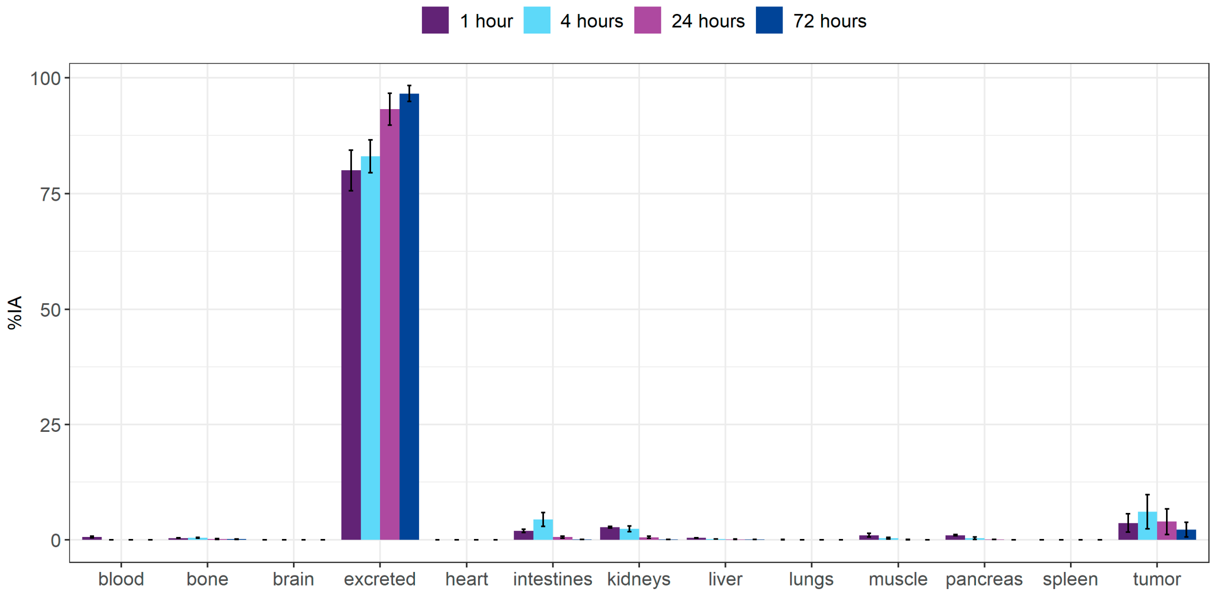

Supplementary Figure S4. Ex vivo biodistribution over time expressed as percentage of injected activity (%IA) of [^{153}Sm]Sm-DOTA-TATE in CA20948 xenograft mice. Values represent mean ± standard deviation (*n* = 3-4).

Supplementary Table S5: Biodistribution data expressed as percentage of injected activity (%IA)* of [^{153}Sm]Sm-DOTA-TATE in CA20498 tumor bearing male NMRI-nude mice. Values represent mean ± standard deviation (*n* = 3-4).

	%ID			
	**1 h**	**4 h**	**24 h**	**72 h**
Blood	0.7 ± 0.2	0.1 ± 0.0	0.0 ± 0.0	0.0 ± 0.0
Bone	0.4 ± 0.0	0.5 ± 0.1	0.2 ± 0.1	0.2 ± 0.0
Brain	0.0 ± 0.0	0.0 ± 0.0	0.0 ± 0.0	0.0 ± 0.0
Excreted	80.0 ± 4.4	83.0 ± 3.5	94.1 ± 3.6	96.6 ± 1.7
Heart	0.0 ± 0.0	0.0 ± 0.0	0.0 ± 0.0	0.0 ± 0.0
Intestines	2.0 ± 0.3	4.4 ± 1.5	0.6 ± 0.2	0.1 ± 0.0
Kidneys	2.8 ± 0.2	2.4 ± 0.6	0.5 ± 0.2	0.1 ± 0.0
Liver	0.4 ± 0.1	0.2 ± 0.0	0.1 ± 0.0	0.2 ± 0.0
Lungs	0.1 ± 0.0	0.0 ± 0.0	0.0 ± 0.0	0.0 ± 0.0
Muscle	1.0 ± 0.4	0.4 ± 0.2	0.1 ± 0.0	0.0 ± 0.0
Pancreas	1.0 ± 0.1	0.4 ± 0.3	0.1 ± 0.0	0.0 ± 0.0
Spleen	0.0 ± 0.0	0.0 ± 0.0	0.0 ± 0.0	0.0 ± 0.0
Stomach	0.7 ± 0.1	1.0 ± 0.3	0.3 ± 0.2	0.1 ± 0.0
Tumor	3.7 ± 2.0	6.1 ± 3.7	3.2 ± 2.9	2.2 ± 1.6

* Percentage of injected activity calculated as (cpm in organ/total cpm) x 100

Supplementary Table S6: Biodistribution data expressed as standard uptake value (SUV)* of [^{153}Sm]Sm-DOTA-TATE in CA20498 tumor bearing male NMRI-nude mice. Values represent mean ± standard deviation (*n* = 3-4).

	SUV			
	**1 h**	**4 h**	**24 h**	**72 h**
Blood	0.1 ± 0.0	0.0 ± 0.0	0.0 ± 0.0	0.0 ± 0.0
Bone	0.1 ± 0.0	0.1 ± 0.0	0.0 ± 0.0	0.0 ± 0.0
Brain	0.0 ± 0.0	0.0 ± 0.0	0.0 ± 0.0	0.0 ± 0.0
Heart	0.0 ± 0.0	0.0 ± 0.0	0.0 ± 0.0	0.0 ± 0.0
Kidneys	1.8 ± 0.1	1.7 ± 0.3	0.3 ± 0.1	0.1 ± 0.0
Liver	0.1 ± 0.0	0.0 ± 0.0	0.0 ± 0.0	0.0 ± 0.0
Lungs	0.1 ± 0.0	0.0 ± 0.0	0.0 ± 0.0	0.0 ± 0.0
Muscle	0.2 ± 0.1	0.1 ± 0.0	0.0 ± 0.0	0.0 ± 0.0
Pancreas	1.2 ± 0.2	0.6 ± 0.4	0.1 ± 0.0	0.0 ± 0.0
Spleen	0.1 ± 0.0	0.1 ± 0.0	0.0 ± 0.0	0.0 ± 0.0
Tumor	4.5 ± 2.0	4.4 ± 1.1	1.9 ± 1.2	1.7 ± 1.0

* SUV = Standardized uptake value. Calculated as (radioactivity in cpm in organ/weight of organ in grams) / total cpm recovered/body weight).
